# Roles of Impulsivity, Motivation, and Emotion Regulation in Procrastination – Path Analysis and Comparison Between Students and Non-students

**DOI:** 10.3389/fpsyg.2018.00891

**Published:** 2018-06-05

**Authors:** Marek Wypych, Jacek Matuszewski, Wojciech Ł. Dragan

**Affiliations:** ^1^Laboratory of Brain Imaging, Nencki Institute of Experimental Biology of Polish Academy of Sciences, Warsaw, Poland; ^2^Faculty of Psychology, University of Warsaw, Warsaw, Poland

**Keywords:** procrastination, impulsivity, emotion regulation, motivation, students and non-students

## Abstract

Procrastination – an irrational delay of intended actions despite expecting to be worse off – is a complex and non-homogenous phenomenon. Previous studies have found a number of correlates of procrastination, some of which seem to be particularly important. Impulsivity is closely connected to procrastination on behavioral, genetic, and neuronal levels. Difficulties in emotion regulation have also been shown to be strongly related to procrastination. Procrastination can also be considered as a motivation-based problem. To try to disentangle the connections of impulsivity, emotion regulation, and motivation to procrastination we collected data from over 600 subjects using multiple questionnaires (PPS – Pure Procrastination Scale; UPPSP – Impulsive Behavior Scale, ERQ – Emotion Regulation Questionnaire and MDT – Motivational Diagnostic Test). Structural equation modeling was performed to test several possible relationships between the measured variables. The effects of student status and age have also been investigated. The final path model was a directional model based on six explanatory variables and accounted for 70% of the variance in procrastination. Path analysis revealed that the strongest contributions to procrastination came from lack of value, delay discounting, and lack of perseverance, suggesting the involvement of motivation and impulsivity. The model also revealed the moderating role of expressive suppression between several aspects of impulsivity and procrastination. Close inspection of the paths’ weights suggests that there may be two partly competing strategies for dealing with impulsivity and negative emotions: either to suppress emotions and impulsive reactions or to react impulsively, discarding previous plans, and to procrastinate. Path invariance analysis showed the significant moderating roles of student status and age. Both in non-students and high-age groups, the path leading from suppression to procrastination was insignificant. This suggests that caution should be used in generalizing the results of studies carried out on students. These results support previous findings that procrastination may serve as a short-term mood regulation strategy. However, as the spectrum of the emotion regulation strategies included in the study was very limited, we conclude that future studies should seek more insight into the relationship between emotion regulation, self-control, and procrastination.

## Introduction

Procrastination – an irrational delay of intended actions despite expecting to be worse off for the delay (cf. Steel, 2007) – is currently considered as a self-regulation failure. Despite numerous studies, procrastination is not yet fully understood and is still regarded as a complex and inhomogeneous phenomenon.

Amongst the many different approaches to procrastination research, previous studies have found a number of its correlates. These include personality traits like impulsivity, neuroticism, and conscientiousness (e.g., Schouwenburg and Lay, 1995; Watson, 2001; meta-analysis and review in Steel, 2007) as well as other constructs including emotion regulation (Tice and Bratslavsky, 2000; Sirois and Pychyl, 2013), stress coping (meta-analysis in Sirois and Kitner, 2015), self-efficacy and motivation (e.g., Steel, 2011), and many others (see van Eerde, 2003; and Steel, 2007 for reviews). In this paper we focus on three factors related to procrastination: impulsivity, emotion regulation, and motivation.

Impulsivity seems to be particularly important for the explanation of mechanisms underlying procrastination. Questionnaire-based research shows a close relationship between these constructs (*r* = 0.41; meta-analysis in Steel, 2007). Within behavioral impulsivity-related measures, procrastinators show deficits in inhibition (Gustavson et al., 2015; Rebetez et al., 2016) and error processing (Michałowski et al., 2017; Wypych et al., 2017). There is also a growing body of evidence showing biological connections between impulsivity and procrastination. First, behavioral genetics studies (Gustavson et al., 2014, 2015; Loehlin and Martin, 2014) have shown that there are common genetic factors. Second, a recent brain structure analysis has shown a negative correlation between procrastination and gray matter volume in the left dorsolateral prefrontal cortex (DLPFC). The left DLPFC was also one of a few brain regions where the same relationship was found for impulsivity (Liu and Feng, 2017).

A recent review by Pychyl and Sirois (2016) emphasizes the role of emotion regulation and stress coping in procrastination. Procrastination was postulated to result from negative emotions, such as fear of failure (Schouwenburg, 1992) or discomfort intolerance (Harrington, 2005). Accordingly, highly procrastinating students were found to be much more sensitive to punishment than their not procrastinating colleagues (Michałowski et al., 2017). Additionally, different negative emotions related to tasks were shown to lead to the avoidance of those tasks (Blunt and Pychyl, 2000). Moreover, emotion regulation training has been shown to reduce procrastination (Eckert et al., 2016). There can be at least two, non-exclusive, mechanisms in which poor emotion regulation increases procrastination. First, as stated in the paper by Tice and Bratslavsky (2000), poor emotion regulation skills seem to undermine self-control in general. Our recent fMRI study found highly procrastinating subjects to have attenuated neuronal activity within the anterior cingulate cortex and right DLPFC (regions involved i.a. in monitoring of performance and behavioral control, respectively) during a task including punishment for errors, suggesting that the context of a possible punishment can impair behavioral control in procrastinators (Wypych et al., 2017). Second, postponing certain tasks or actions can be seen as a strategy for short-term mood repair (e.g., Sirois and Pychyl, 2013). Either way, negative emotions seem to amplify procrastination and can lead, ironically, to more stress and more negative emotions and thus more procrastination.

To initiate or finish a task we have to take actions and this requires motivation. Thus, procrastination can also be considered as a motivation-based problem. This point of view has been presented in papers by Steel and König (2006) and Steel (2007) describing the most recent and general theory of motivation: Temporal Motivation Theory (TMT). This theory defines motivation as proportional to people’s self-efficacy (or expectation that efforts will be rewarded) and their feeling of value (meaningfulness or enjoyableness) of certain work. At the same time, motivation is inversely proportional to impulsivity (understood as susceptibility to temptation, or temporal discounting tendency). Motivation is also inversely proportional to time left to the deadline (Steel and König, 2006; Steel, 2007). The TMT and the corresponding Motivational Diagnostic Test (Steel, 2011) seem to be appropriate and effective in explaining procrastination (Steel, 2011) and are strong candidates for further procrastination research.

For practical reasons, the majority of psychological studies, including most procrastination research, involve student subjects. To our knowledge, there are very few procrastination studies published that directly compare students and non-students. In one of these studies Svartdal et al. (2016) showed invariance between students and employees, related mostly to timeliness, in the three-factor model of procrastination. They also showed, that students tend to procrastinate more. In the recent study by Steel et al. (2018) it was shown that correlation between conscious attention control and procrastination is more negative in students. It is already also known that the prevalence of problematic procrastination differs between these populations and is estimated to be about 20% in the general population (e.g., Harriott and Ferrari, 1996) and up to 50% in students (e.g., Solomon and Rothblum, 1984). One reason could be that students often take their obligations and commitments more lightly, as the possible consequences of failures are usually less severe. The differences might be also age-related. Over the course of our lives, we learn and gain experience and it is known that procrastination decreases with age (e.g., the meta-analysis in Steel, 2007). The behavioral differences could also be a result of differences in maturation of the prefrontal cortex as its myelination is not complete at the age of a typical student (e.g., Sowell et al., 1999). The question arises as to whether the mechanisms underlying procrastination in students or younger people are similar to those in general population and to what extent the results and conclusions obtained in students can be generalized.

In this paper we focus on the roles of impulsivity, emotion regulation strategies, and motivation in procrastination. To try to disentangle connections between the measures, we prepared an internet survey and collected data using multiple questionnaires including: PPS – Pure Procrastination Scale (Steel, 2010); UPPSP – Impulsive Behavior Scale (Cyders et al., 2007), ERQ – Emotion Regulation Questionnaire (Gross and John, 2003) and MDT – Motivational Diagnostic Test (Steel, 2011). We then performed structural equation modeling considering a few possible models. We start with a basic model where the variables are freely intercorrelated. However, as stated above, we assume that procrastination can arise as a result of the other factors. Thus we also test a model with paths directed to procrastination. Dewitte and Schouwenburg (2002) suggested that the relationship between several aspects of impulsivity and procrastination may be mediated by other variables (they considered the impact of personality traits). Since it has been shown that suppression of emotion can undermine self-control (e.g., Vohs and Heatherton, 2000) here we propose a model where the emotion regulation mediates between impulsivity and procrastination. Additionally, we inspect whether and how student status and age can influence the relationships between the analyzed variables in the final model.

## Materials and Methods

### Data Collection

Data were collected via online questionnaires on a specially prepared website. As a reward for participation in the study, subjects were prompted to download a 40-page pdf e-book “The bases of effective self-management” by MW. Information about the study was spread mostly via internet social media.

After demographic questions (age, sex, work status, and student status) subjects were given four questionnaires PPS, UPPSP ERQ, and MDT (see below for details) in an order randomly assigned for each participant.

### Subjects

Altogether 979 subjects started to fill in the questionnaires, of which 334 did not complete all the scales needed for our analyses. Further, four subjects were excluded because their ages were below 18. We used the log-likelihood distance influence measure to identify potentially influential cases (Weisberg and Cook, 1982). Although there are no fixed cutoff values for determining the high-influence case, we identified three cases whose influence values were outliers regarding the distribution of the log-likelihood distance influence measure. These three cases were excluded from all further analyses. Analyses were performed on data collected from 638 subjects (476 women, age *M* = 31.16; *SD* = 8.95, see **Table 1** for details). Of those 283 were students (208 women, age *M* = 25.94; *SD* = 5.47) and 355 non-students (268 women, age *M* = 35.32; *SD* = 9.01). We also subdivided the studied group into two age groups based on median age. The first group, aged 18-28 (*M* = 24.13; *SD* = 2.59) included 300 participants (225 women). The second group, aged 29–68 (*M* = 37.4; *SD* = 7.89), included 338 participants (251 women). The row data can be find in the Supplementary Materials.

**Table 1:** Means and SDs (in brackets) of all collected data.

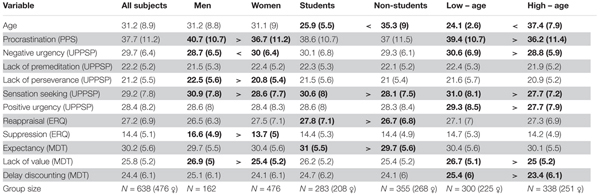

*The values were statistically compared (with t-tests) between men and women, students and non-students and between low- and high age groups. Significant differences (p < 0.05) are marked with bold font and appropriate inequality sign.*

### Measures

#### Pure Procrastination Scale (PPS)

The Pure Procrastination Scale (Steel, 2010, in Polish version by Stępień and Topolewska, 2014) is a one-scale questionnaire measuring Procrastination. Internal consistency of the adapted Polish version is good with Cronbach’s alpha = 0.89 (Stępień and Topolewska, 2014).

#### Impulsive Behavior Scale (UPPSP)

The Impulsive Behavior Scale (Whiteside and Lynam, 2001; Cyders et al., 2007; Polish version: Poprawa, 2014) is a questionnaire of behavioral impulsivity consisting of five scales: Negative Urgency (impulsive behaviors in negative emotion situations), Lack of Premeditation (or lack of planning), Lack of Perseverance, Sensation seeking, and Positive Urgency (impulsive behaviors in positive emotion situations). The Cronbach’s alphas for the scales of the Polish versions are 0.85, 0.84, 0.83, 0.83, and 0.93, respectively, (Poprawa, 2014).

#### Emotion Regulation Questionnaire (ERQ)

The Emotion Regulation Questionnaire (Gross and John, 2003, Polish version by Kobylińska^1^) is a questionnaire assessing the habitual use of two emotion regulation strategies: cognitive Reappraisal (reinterpretation of an emotional situation to alter its meaning and change the emotional impact) and expressive Suppression (attempts to hide and/or reduce ongoing emotions; Gross and John, 2003). The Cronbach’s alphas for the scales of the Polish versions are 0.75 and 0.85, respectively (Śmieja et al., 2011).

#### Motivational Diagnostic Test (MDT)

The Motivational Diagnostic Test (Steel, 2011, Polish translation by Suchodolska, Dragan, Wypych) is a three-scale test, related to TMT, measuring: Expectancy of results of one’s efforts (or self-efficacy), Lack of Value (or meaningfulness and enjoyableness) of one’s work, and tendency of Delay Discounting (or susceptibility to temptation). The last scale was interpreted by the author as “impulsiveness” (Steel, 2011), however, a representative item from the scale is: “*My actions and words satisfy my short-term pleasures rather than my long-term goals.*” In this paper we understand impulsivity as a broader construct and we will refer to this sub-scale as “Delay Discounting.” Internal reliability in this sample was good: Cronbach’s alphas had values of 0.88, 0.8, and 0.86, respectively.

### Statistical Analyses

Our analytic strategy consisted of three steps. First, we performed correlation and partial correlation analyses in order to identify the model of relations between measured variables. We selected specific measures on the basis of their partial correlation with procrastination measured with the PPS and theoretical assumptions. Subsequently, we performed a series of SEM analyses to explore relationships between variables selected in the previous step. A baseline model was constructed with latent factors based on manifest items freely intercorrelated. The fit of subsequent modifications of this model was compared to the best-fit model (at a given stage of analysis) on the basis of χ^2^ statistics. In order to examine the moderating role of the group (students vs. non-students and low age vs. high age), during the third step of the analysis we examined the final model based on Jöreskog (1971) method for the comparison of models in different populations.

Descriptive statistics, as well as correlations and partial correlations, were calculated using MATLAB R2017b (Mathworks Inc.). Structural Equation Modeling was carried out using Mplus 7 (Muthén and Muthén, 2012). We used a maximum likelihood estimator. Model fit was evaluated using various criteria including the chi-square test, the root mean square error of approximation (RMSEA), the standardized root mean residual (SRMR), the comparative fit index (CFI), and the Tucker-Lewis index (TLI) and was considered good when it fulfilled the criteria: RMSEA < 0.06, SRMR < 0.08, CFI and TLI both >0.95 (Hu and Bentler, 1999). Bootstrapping was used to calculate the 95% confidence intervals of path coefficients (5000 resamples were taken for these analyses).

## Results

### Descriptive Statistics

Descriptive statistics of all collected measures for the whole sample as well as for men, women, students, non-students, and low and high age subsamples are presented in **Table 1**, and correlations and partial correlations between the variables are presented in **Table 2**. For computing of partial correlations, the Sensation Seeking scale was excluded as it did not correlate with PPS (*r* = -0.05 *p* = 0.23).

**Table 2:** Pearson’s correlations (above diagonal) and partial correlations (below diagonal, italic) between collected demographic and questionnaire measures.

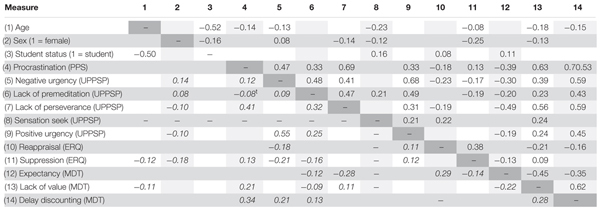

*Non-significant correlations (p > 0.05) were omitted. Sensation Seeking was not included while computing the partial correlations. ^*t*^ in the partial correlation between Lack of Premeditation and Procrastination denotes a trend with p = 0.053.*

### Best-Fitted SEM Model Identification

We began the identification of best-fit SEM models of relationships between measured variables by the construction of a CFA model with latent factors freely intercorrelated. Using the results of partial correlation analysis (zero or non-significant relations with PPS) we excluded the following scales from further analysis: Sensation Seeking and Positive Urgency (UPPSP), Reappraisal (ERQ), and Expectancy (MDT). Items of the remaining scales (UPPSP: Negative Urgency, Lack of Premeditation and Lack of Perseverance; ERQ: Suppression; MDT: Lack of Value and Delay Discounting) built latent factors in the baseline CFA model (Model 1). Note, however, that partial correlation between Lack of Premeditation and PPS was on a trend level (*r* = -0.08, *p* = 0.053). The fit statistics of this model are presented in **Table 3**. The baseline model fit was unsatisfactory.

**Table 3:** Comparison between different models - model fit statistics.

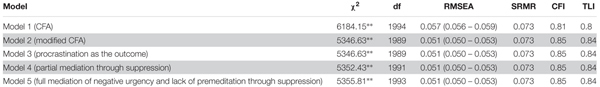

**RMSEA, the root mean square error of approximation; SRMR, the standardized root mean residual; CFI, the comparative fit index; TLI, the Tucker–Lewis index; the 90% CI for RMSEA are in brackets; df, degrees of freedom; ^∗∗^p < 0.001.**

To improve the fit statistics of the model we allowed for correlations between the error terms of five sets of items: item 11 and item 20 of MDT, item 17 and item 23 of MDT, item 1 and item 2 of PPS, item 10 and item 11 of PPS, and item 11 and item 12 of PPS. Item 11 of MDT (“Work bores me”) and item 20 of MDT (“I don’t find my work enjoyable”) share the same content – the attitude toward work. Item 17 of MDT (“When a task is tedious, again and again I find myself pleasantly daydreaming rather than focusing”) and item 23 of MDT (“If an activity is boring, my mind slips off onto other diversions”) are both related to redirecting the attention to external cues. Item 1 of PPS (“I delay making decisions until it’s too late”) and item 2 of PPS (“Even after I make a decision I delay acting upon it”) concern the same topic – delaying decisions. Furthermore, item 10 of PPS (“I don’t get thing done on time”), item 11 of PPS (“I am not very good at meeting deadlines”), and item 12 of PPS (“Putting things off till the last minute has cost me the money in the past”) share the same content – the idea of keeping the deadline. The correlations between error terms appear to represent an underlying theoretically and empirically meaningful relation between the items involved. Hence, there is sufficient rationale for including correlations between error terms of the five sets of items in the model. The modified CFA model fit was more acceptable (the fit statistics are presented in **Table 3**). RMSEA and SRMR met the threshold for good fit, and CFI and TLI were below this threshold but still on the level, which is considered as reasonable. The comparison of models 1 and 2 using the chi-square difference was significant – Δχ^2^(*df* = 5) = 837.52, *p* < 0.001. This finding indicates that model 2 yields a better fit than model 1.

In the next step, we tested an alternative model in which we allowed for direct paths from all latent factors representing different aspects of impulsivity, motivation problems, and emotion regulation strategies to procrastination (model 3). The fit statistics of this model (as shown in **Table 3**) were the same as model 2. It indicates that directional character of the relations between impulsivity, motivation and emotion regulation, and procrastination, cannot be excluded. Given our theoretical predictions that procrastination is the outcome rather than the cause of impulsivity and difficulties in emotion regulation, and motivation, we decided to set model 3 as the baseline in further steps.

Subsequently, we decided to compare fit statistics of model 3 and its modification in which we added mediation paths from Negative Urgency, Lack of Perseverance, and Delay Discounting to Procrastination through Suppression (simultaneously keeping the direct paths). We decided to test mediation of Delay Discounting due to its strong theoretical bonds with impulsiveness (Steel, 2011). The fit statistics of model 4 (as shown in **Table 3**) were acceptable (see “*Concluding remarks and limitations of the study*” section). The comparison of models 3 and 4 using the chi-square difference was insignificant – Δχ^2^(*df* = 2) = 5.8, *p* > 0.05. This finding indicates that both models fit equally well and the more restricted model 4 should be favored.

In next step, we decided to remove insignificant paths from Negative Urgency and Lack of Perseverance to Procrastination and test the model in which these two traits are fully mediated by Suppression (model 5). The fit statistics of model 5 were acceptable. The comparison between models 4 and 5 using the chi-square difference was insignificant – Δχ^2^(*df* = 2) = 3.38, *p* > 0.05. This finding indicates that both models fit equally well and the more restricted model 5 should be favored. This model was established as the final one.

**FIGURE 1:**
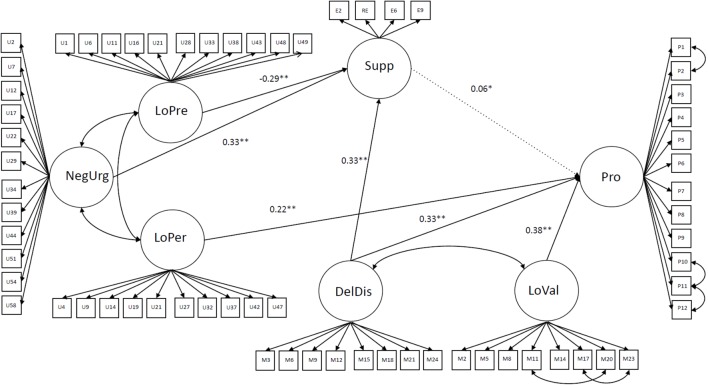
The final path model of impulsivity, motivation, and emotion regulation with procrastination. The model was obtained for the whole sample. Pro, procrastination; Supp, suppression; DelDis, delay discounting; LoVal, lack of value; NegUrg, negative urgency; LoPre, lack of premeditation; LoPer, lack of perseverance; P1–P12, items of Pure Procrastination Scale; E2–E9, items of Emotion Regulation Questionnaire; U1–U58, items of UPPSP; M2 – M24, items of Motivational Diagnostic Scale, ^∗∗^*p* < 0.001, ^∗^*p* < 0.01. Path marked with dashed line was insignificant in the non-student and high-age subgroups, see text for details. Errors of observed variables and correlation paths between MDT traits and UPPSP traits were omitted for clarity.

The results for the final path model (model 5) with standardized coefficients are presented in **Figure 1** (unstandardized coefficients with 95% confidence intervals for all paths are and the Mplus script of the final model are presented in the Supplementary Materials). This model accounted for 70% of the variance in procrastination. Higher intensity of Lack of Perseverance, Delay Discounting, Lack of Value and Suppression was related to the higher intensity of Procrastination. Increase in Negative Urgency and Delay Discounting was related to an increase in Suppression. However, the latter was negatively associated with Lack of Premeditation.

Indirect effects of Delay discounting, Negative Urgency and Lack of Premeditation on procrastination were not significant (β = 0.02, 95%*CI*: 0.01–0.4, *p* < 0.1, β = 0.02, 95%*CI*: 0.01–0.4, *p* < 0.1 and β = -0.01, 95%*CI*: -0.02–0, *p* > 0.1, respectively).

### Multiple Group Model Comparison

Finally, we tested the possible moderating role of the group (students vs. non-students). The final model adequately fits both groups (as shown in **Table 4**). Thus, we performed a fit analysis of the unconstrained final model using a group-weighted sample. The fit statistics are presented in **Table 4**. Finally, we tested a model in which all pathways were constrained to be equal across both groups. Again, this model fits well (all fit statistics are shown in **Table 4**). Comparison of the unconstrained and constrained models using the chi-square difference was significant -Δχ^2^(*df* = 12) = 23.5, *p* < 0.05. This implies that the path coefficients across groups are not equal and that the student status may be a moderator of the relationship between measured variables. A close examination of path coefficients in both groups revealed that the path from Suppression to Procrastination is not significant in the non-student group (β = -0.01, *p* > 0.05 vs. β = 0.07, *p* < 0.01 in student group). Additionally, we tested the indirect effect of Delay Discounting, Negative Urgency and Lack of Premeditation on procrastination in the student group. The indirect effect was significant only for Negative Urgency (β = 0.04, 95%CI: 0.01–0.07, *p* < 0.05). Indirect effects of Delay Discounting and Lack of Premeditation were not significant (β = 0.03, 95%*CI*: 0–0.06, *p* < 0.05; and β = -0.03, 95%*CI*: -0.06–0, *p* < 0.05, respectively).

**Table 4:** Multiple group (students vs. non-students and low-age vs. high age groups) comparisons for Model 5 – model fit statistics.

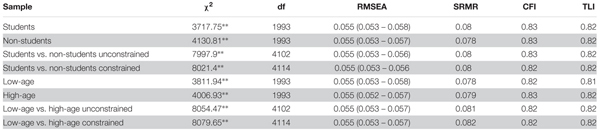

**RMSEA, the root mean square error of approximation; SRMR, the standardized root mean residual; CFI, the comparative fit index; TLI, the Tucker–Lewis index; the 90% CI for RMSEA are in brackets; df, degrees of freedom; ^∗∗^p < 0.001.**

Taking into account that students and non-students differed in age, we checked whether the result is not an effect of these differences. The final model fits adequately in both age groups. Again, we tested the unconstrained final model using a group-weighted sample. In the next step, we tested a model in which all pathways were constrained to be equal across both age groups (fit statistics of the all models are presented in **Table 4**). Comparison of the unconstrained and constrained models using the chi-square difference was significant -Δχ^2^(*df* = 12) = 25.18, *p* < 0.05. The examination of path coefficients in both age groups revealed that the path from Suppression to Procrastination is not significant in the high age group (β = 0.01, *p* > 0.05 vs. β = 0.1, *p* < 0.05 in the low age group).

Finally, we tested indirect effect of Delay Discounting, Negative Urgency and Lack of Premeditation on Procrastination in the low age group. For all three traits the indirect effect was not-significant (β = 0.02, 95%*CI*: 0–0.05, *p* > 0.05; β = 0.035, 95%*CI*: 0–0.07, *p* > 0.05 and β = -0.02, 95%*CI*: -0.05–0, *p* > 0.05, respectively).

Unfortunately, the small number of men in our sample did not allow for analogous comparison between genders.

## Discussion

In this work, we aimed to add new insights to our understanding of the roles of impulsivity, emotion regulation strategies, and motivation in procrastination. To do this, we collected and analyzed questionnaire data addressing those constructs. While analyzing the data, we also divided our sample into subsamples to look for potential differences between students and non-students as well as between low-age and high-age subgroups.

### Procrastination and Demography

Differences in procrastination related to demography can be seen from the descriptive statistics and the correlation matrix. First, the correlation of *r* = -0.14 between age and procrastination is very similar to what has been reported in the literature previously (e.g., Steel and Ferrari, 2013), suggesting that with age and experience we improve slightly in self-control or that we provide more desirable responses. Consistent with previous research, men in our sample procrastinate significantly more than women (*r* = -0.16 for the whole sample, cf. *r* = -0.08 in Steel and Ferrari, 2013). This result goes together with significantly lower perseverance and perceived value of work (i.e., higher scores in the lack of them) and higher suppression in men, suggesting a possible contribution of all three factors: impulsivity, emotion regulation, and motivation. We did not find a significant difference in procrastination related to student status.

### Path Model of Impulsivity, Emotion Regulation, and Motivation in Procrastination

The final, directional, path model was built based on six explanatory variables and accounted for 70% of the variance in procrastination.

First, the most robust direct paths to procrastination were from lack of value, delay discounting, and lack of perseverance. The substantial input from lack of value implies that low motivation and/or weak sense of the meaning of the undertaken efforts can undermine our actions. The contribution of lack of perseverance to procrastination is not surprising, as the scales are related almost by their definitions. Delay discounting from MDT was interpreted by its author (Steel, 2011) as impulsiveness but seems to capture additional aspects not fully covered by UPPSP. The high tendency for temporal discounting in procrastinators has already been experimentally proven (Wu et al., 2016). Together these results are consistent with TMT by Steel and König (2006).

Suppression seems to mediate between several aspects of impulsivity (lack of premeditation, negative urgency, and delay discounting) and procrastination. However, the weight of the path between suppression and procrastination is surprisingly low. Moreover, the indirect effects of these variables on procrastination were not significant. Together with the strong direct inputs to procrastination from delay discounting and lack of perseverance, this could suggest that procrastination and suppression serve as partly complementary mechanisms for dealing with impulsivity and negative emotions. Some people may tend to react more impulsively (e.g., discard previous plans) and others to suppress emotions and impulsive reactions. However, it is also possible that the choice between both strategies is realized on the individual level. Particular emotion regulation strategies are not always beneficial and sometimes people need to switch to another one (see for example Bonanno and Burton, 2013).

Interestingly, the weight of the path between lack of premeditation or planning and suppression is negative. This suggests that suppression and planning are positively related. This could mean that both suppression and planning represent attempts to take control over one’s actions. Yet there was no indirect effect of lack of premeditation on procrastination. This is, however, consistent with previous research indicating that increased planning and time management interventions did not decrease procrastination in the long term (see review in van Eerde, 2015, and meta-analysis in van Eerde, 2017). Interestingly, the weight of path between negative urgency and suppression is positive. One might expect that, as negative urgency represents impulsive reactions to negative emotions, it should be negatively related to expressive suppression. However, a positive relation between both constructs was recently found in gamblers (Navas et al., 2017), suggesting a more complex relationship between negative urgency and suppression.

Based on current knowledge on the relationship between procrastination and emotion regulation and/or coping (e.g., Blunt and Pychyl, 2000; Harrington, 2005, reviews in Sirois and Pychyl, 2013; Pychyl and Sirois, 2016), one might expect that less adaptive emotion regulation strategies should be strong predictors of procrastination. However, the emotion regulation measure used in this study (ERQ) consists only of two scales: reappraisal and suppression, the first of which is believed to be adaptive and the second, maladaptive. In fact, ERQ does not include a broader spectrum of maladaptive strategies (e.g., distraction). Negative urgency, although formally belonging to the impulsivity measure (UPPSP), could also be interpreted as a mechanism for coping with negative emotions. However, the path directed from negative urgency to procrastination in our model was also not significant. The relationship between emotion regulation skills and/or stress coping and procrastination requires further investigation.

### Group Differences

Path invariance analysis revealed the significant moderating role of the group, both in students vs. non-students as well as low-age vs. high-age (cf. Svartdal et al., 2016). The pathway directed from suppression to procrastination was significant only in the student and the low-age subgroups and insignificant in the non-student and the high-age subgroups. This finding suggests that age rather than student status is a cause of this difference and that suppression mediates between impulsivity and procrastination only at a young age. This could be related to more emotional clarity gained with age and experience and/or with the development with age of healthier strategies (John and Gross, 2004) including a decrease in suppression (Zimmermann and Iwanski, 2014). This result could also to go in line with the finding of Steel et al. (2018) that correlation between conscious attention control and procrastination is more negative in students. On the other hand, the only significant indirect effect in this analysis was found for negative urgency in the student group. This suggests that impulsive reactions to negative emotions can, indirectly, result in procrastination in students. Our results suggest that, with age and experience, procrastination and suppression could constitute two separate strategies for dealing with impulsivity and/or negative emotions. We also conclude, that, despite seemingly subtle between-group differences, caution is required when generalizing results obtained from student populations.

### Concluding Remarks and Limitations of the Study

In this study, we tried to disentangle the roles of impulsivity, emotion regulation strategies, and motivation in procrastination. Apparently, all three constructs underlie procrastinatory behaviors. However, from variables included in our study, lack of perseverance, delay discounting, and lack of value seem to be the most important.

Our study has several limitations. First, the sample size and particularly a low number of male subjects, suggests caution in the generalization of the results. Second, not all the fit statistics of the final model met the thresholds indicated by Hu and Bentler (1999) for good fit. However, as pointed by Kenny (2014) incremental fit indices such as CFI and TLI may not be thoroughly informative if the RMSEA for the null model is less than 0.158 (in the case of our study null RMSEA for the final model was 0.128). Third, the spectrum of the emotion regulation strategies included in the study was very limited. Previous research suggests that procrastination may be a result of weakened self-control in the presence of negative emotions, but also that it can serve as an emotion regulation strategy itself. Future studies should attempt to delve into the relationship between emotion regulation, self-control, and procrastination.

## Ethics Statement

The protocol of the study was approved by the Ethics Committee of the Faculty of Psychology at the University of Warsaw in accordance with the Declaration of Helsinki.

## Author Contributions

MW plan and organization of the research, data analysis, and manuscript writing. JM and WD data analysis and manuscript writing.

## Conflict of Interest Statement

The authors declare that the research was conducted in the absence of any commercial or financial relationships that could be construed as a potential conflict of interest.

## Footnotes

1 https://spl.stanford.edu/sites/default/files/polish.pdf
