# Supplementary material for: Roles of Impulsivity, Motivation, and Emotion Regulation in Procrastination – Path Analysis and Comparison Between Students and Non-students
Source: Front Psychol. 2018 Jun 5;9:891. doi: 10.3389/fpsyg.2018.00891 (PMC5996249; doi:10.3389/fpsyg.2018.00891)
Supplement: Supplementary file 1 [file Table_1.pdf]

Unstandardized coefficients with 95% confidence intervals for all paths in the final SEM model. "E" denotes items of ERQ, "M" refers to MDT, "P" refers to items of PPS and "U" denotes items of UPPSP. See scales description for details.

Path	Unstandardized coefficients with 95% CI
Suppression BY	
E2	1
E4	0.52 (95%CI: 0.44 – 0.6); p<0.001
E6	1 (95%CI: 0.91 – 1.11); p<0.001
E9	0.85 (95%CI: 0.76 – 0.94); p<0.001
Lack of Value BY	
M2	1
M5	0.23 (95%CI: 0.13 – 0.35); p<0.001
M8	0.94 (95%CI: 0.79 – 1.12); p<0.001
M11	1.1 (95%CI: 0.92 – 1.29); p<0.001
M14	1.58 (95%CI: 1.38 – 1.83); p<0.001
M17	1.07 (95%CI: 0.94 – 1.23); p<0.001
M20	1.03 (95%CI: 0.84 – 1.23); p<0.001
M23	0.94 (95%CI: 0.82 – 1.09); p<0.001
Delay Discounting BY	
M3	1
M6	0.58 (95%CI: 0.49 – 0.69); p<0.001
M9	0.79 (95%CI: 0.71 – 0.88); p<0.001
M12	0.96 (95%CI: 0.87 – 1.06); p<0.001
M15	0.9 (95%CI: 0.82 – 0.99); p<0.001
M18	0.92 (95%CI: 0.83 – 1.01); p<0.001
M21	0.99 (95%CI: 0.91 – 1.08); p<0.001
M24	0.87 (95%CI: 0.78 – 0.95); p<0.001
Procrastination (PPS) BY	
P1	1
P2	1.09 (95%CI: 1.02 – 1.18); p<0.001
P3	1.09 (95%CI: 1 – 1.18); p<0.001
P4	1.17 (95%CI: 1.06 – 1.29); p<0.001
P5	1.29 (95%CI: 1.17 – 1.42); p<0.001
P6	1.22 (95%CI: 1.11 – 1.35); p<0.001
P7	1.29 (95%CI: 1.17 – 1.41); p<0.001
P8	1.27 (95%CI: 1.16 – 1.39); p<0.001
P9	1.16 (95%CI: 1.05 – 1.27); p<0.001
P10	1.12 (95%CI: 1.02 – 1.24); p<0.001
P11	1.13 (95%CI: 1.03 – 1.23); p<0.001
P12	1.04 (95%CI: 0.94 – 1.15); p<0.001
Negative Urgency BY	
U2	1
U7	0.79 (95%CI: 0.66 – 0.93); p<0.001
U12	0.92 (95%CI: 0.8 – 1.05); p<0.001

U17	1.09 (95%CI: 0.96 – 1.26); p<0.001
U22	0.78 (95%CI: 0.65 – 0.94); p<0.001
U29	1.12 (95%CI: 1 – 1.26); p<0.001
U34	1.22 (95%CI: 1.08 – 1.39); p<0.001
U39	1.01 (95%CI: 0.89 – 1.14); p<0.001
U44	1.25 (95%CI: 1.12 – 1.4); p<0.001
U51	1.06 (95%CI: 0.93 – 1.2); p<0.001
U54	-0.93 (95%CI: -1.06 – -0.78); p<0.001
U58	1.12 (95%CI: 1.01 – 1.26); p<0.001
Lack of Premeditation BY	
U1	1
U6	1.01 (95%CI: 0.92 – 1.11); p<0.001
U11	-0.67 (95%CI: -0.82 – -0.51); p<0.001
U16	1.19 (95%CI: 1.05 – 1.33); p<0.001
U21	-0.93 (95%CI: -1.07 – -0.81); p<0.001
U28	1.16 (95%CI: 1.04 – 1.28); p<0.001
U33	1.06 (95%CI: 0.96 – 1.19); p<0.001
U38	0.93 (95%CI: 0.86 – 1.06); p<0.001
U43	0.79 (95%CI: 0.68 – 0.91); p<0.001
U48	1.21 (95%CI: 1.08 – 1.35); p<0.001
U49	1.17 (95%CI: 1.04 – 1.31); p<0.001
Lack of Persistence BY	
U4	1
U9	-0.84 (95%CI: -0.98 – -0.71); p<0.001
U14	0.5 (95%CI: 0.4 – 0.6); p<0.001
U19	0.96 (95%CI: 0.85 – 1.09); p<0.001
U24	0.87 (95%CI: 0.75 – 1.01); p<0.001
U27	1.28 (95%CI: 1.17 – 1.42); p<0.001
U32	1.03 (95%CI: 0.9 – 1.19); p<0.001
U37	1.36 (95%CI: 1.22 – 1.54); p<0.001
U42	1.21(95%CI: 1.09 – 1.28); p<0.001
U47	-0.98 (95%CI: -1.13 – -0.84); p<0.001
Procrastination (PPS) ON	
Lack of Persistence	0.33 (95%CI: 0.19 – 0.49); p<0.001
Delay Discounting	0.31 (95%CI: 0.21 – 0.42); p<0.001
Lack of Value	0.52 (95%CI: 0.35 – 0.73); p<0.001
Suppression	0.03 (95%CI: 0 – 0.06); p<0.1
Suppression ON	
Lack of Premeditation	-0.62 (95%CI: -0.9 – -0.35); p<0.001
Negative Urgency	0.91 (95%CI: 0.58 – 1.27); p<0.001
Delay Discounting	0.55 (95%CI: 0.37 – 0.74); p<0.001
Delay Discounting WITH	
Lack of Value	0.35 (95%CI: 0.3 – 0.42); p<0.001
Negative Urgency WITH	
Delay Discounting	-0.13 (95%CI: -0.16 – -0.1); p<0.001
Lack of Value	-0.25 (95%CI: -0.3 – -0.21); p<0.001

Lack of Premeditation WITH	
Lack of Value	0.06 (95%CI: 0.04 – -0.21); p<0.001
Delay Discounting	0.16 (95%CI: 0.13 – 0.2); p<0.001
Negative Urgency	-0.1 (95%CI: -0.13 – -0.08); p<0.001
Lack of Persistence WITH	
Lack of Value	0.2 (95%CI: 0.17 – 0.23); p<0.001
Delay Discounting	0.27 (95%CI: 0.23 – 0.32); p<0.001
Negative Urgency	-0.1 (95%CI: -0.13 – -0.08); p<0.001
Lack of Premeditation	0.11 (95%CI: 0.09 – 0.14); p<0.001
M11 WITH	
M20	0.47 (95%CI: 0.4 – 0.56); p<0.001
M17 WITH	
M23	0.26 (95%CI: 0.2 – 0.32); p<0.001
P10 WITH	
P11	0.53 (95%CI: 0.46 – 0.6); p<0.001
P11 WITH	
P12	0.19 (95%CI: 0.12 – 0.25); p<0.001
P1 WITH	
P2	0.26 (95%CI: 0.2 – 0.32); p<0.001
