# Supplementary material for: Roles of Impulsivity, Motivation, and Emotion Regulation in Procrastination – Path Analysis and Comparison Between Students and Non-students
Source: Front Psychol. 2018 Jun 5;9:891. doi: 10.3389/fpsyg.2018.00891 (PMC5996249; doi:10.3389/fpsyg.2018.00891)
Supplement: Supplementary file 2 [file Presentation_1.pdf]

Subscales of the questionnaires used in the study and items building the subscales.

1. ERQ scales:

Reappraisal = erq_1 + erq_3 + erq_5 + erq_7 + erq_8+ erq_10
Suppression = erq_2+ erq_4 + erq_6 + erq_9

2. MDT scales:

Expectancy = mdt_1 + mdt_4 + mdt_7 + mdt_10 + mdt_13 + mdt_16 + mdt_19+ mdt_22
Value = mdt_2 + mdt_5 + mdt_8 + mdt_11 + mdt_14 + mdt_17 + mdt_20 + mdt_2
Delay = mdt_3 + mdt_6 + mdt_9 + mdt_12 + mdt_15 + mdt_18 + mdt_21 + mdt_24

3. PPS score:

pps_1 + pps_2 + pps_3 + pps_4 + pps_5 + pps_6 + pps_7 + pps_8 + pps_9 + pps_10 + pps_11 + pps_12

4.UPPSP scales:

Reverse-scoring items (1 -> 4, 2 -> 3, 3 ->2, 4 -> 1):

upps_2 upps_7 upps_12 upps_17 upps_22 upps_29 upps_34 upps_39 upps_44 upps_51 upps_58 upps_11 upps_21 upps_9 upps_47 upps_3 upps_8 upps_13 upps_18 upps_23 upps_26 upps_31 upps_36 upps_41 upps_46 upps_52 upps_56 upps_5 upps_10 upps_15 upps_20 upps_25 upps_30 upps_35 upps_40 upps_45 upps_50 upps_53 upps_55 upps_57 upps_59

Negative urgency (r = reversed item)**:** upps_2r + upps_7r+ upps_12r + upps_17r + upps_22r + upps_29r + upps_34r + upps_39r + upps_44r + upps_51r + upps_54 + upps_58r
Premeditation (r = reversed item)**:** upps_1 + upps_6 + upps_11r + upps_16 + upps_21r + upps_28 + upps_33 + upps_38 + upps_43 + upps_48 + upps_49
Perseverance (r = reversed item)**:** upps_4 + upps_9r + upps_14 + upps_19 + upps_24 + upps_27 + upps_32 + upps_37 + upps_42 + upps_47r
Sensation seeking (r = reversed item)**:** upps_3r + upps_8r + upps_13r + upps_18 r + upps_23r + upps_26r + upps_31r + upps_36r + upps_41r + upps_46r + upps_52r + upps_56r
Positive Urgency (r = reversed item)**:** upps_5r + upps_10r + upps_15r + upps_20r + upps_25r + upps_30r + upps_35r + upps_40r + upps_45r + upps_50r + upps_53 r+ upps_55r + upps_57r + upps_59r
