# Supplementary material for: Roles of Impulsivity, Motivation, and Emotion Regulation in Procrastination – Path Analysis and Comparison Between Students and Non-students
Source: Front Psychol. 2018 Jun 5;9:891. doi: 10.3389/fpsyg.2018.00891 (PMC5996249; doi:10.3389/fpsyg.2018.00891)
Supplement: Supplementary file 3 [file Presentation_2.pdf]

MPlus script of the final model obtained in the analysis.

```
TITLE : Procrastination - final mediation model

DATA:
        ! here enter the name and the path of the data set
        FILE =

VARIABLE:
        ! e - ERQ questionnaire items
        ! p - PPS questionnaire items
        ! u - UPPSP questionnaire items
        ! m - MDT questionnaire items

        NAMES ARE kod age sex student
        e1-e10 m1-m24 p1-p12 u1-u59;
        USEVARIABLES = e2 e4 e6 e9
        m2 m3 m5 m6 m8 m9 m11 m12 m14
        m15 m17 m18 m20 m21 m23 m24
        p1-p12 u1 u2 u4 u6 u7 u9 u11 u12
        u14 u16 u17 u19 u21 u22 u24 u27-u29
        u32-u34 u37-u39 u42-u44 u47-u49 u51
        u54 u58;

ANALYSIS:
        TYPE = GENERAL;
        ESTIMATOR = ML;

MODEL:

        !SUP - Suppresion
        !VAL - Lack of Value
        !DEL - Delay Discounting
        !PPS - Procrastination
        !NU - Negative Urgency
        !PRE - Premeditation
        !PER - Lack of Perseverance
        SUP BY e2 e4 e6 e9;
        VAL BY m2 m5 m8 m11 m14 m17 m20 m23;
        DEL BY m3 m6 m9 m12 m15 m18 m21 m24;
        PPS BY p1-p12;
        NU BY u2 u7 u12 u17 u22 u29 u34 u39 u44 u51 u54 u58;
        PRE BY u1 u6 u11 u16 u21 u28 u33 u38 u43 u48 u49;
```

```
    PER BY u4 u9 u14 u19 u24 u27 u32 u37 u42 u47;
    m11 WITH m20;
    m17 WITH m23;
    p10 WITH p11;
    p11 WITH p12;
    p1 WITH p2;
    PPS ON PER DEL VAL SUP PRE NU;
    SUP ON PRE NU DEL;

OUTPUT:
    tech1;
```
